# Decreased brain activity within the dorsolateral prefrontal cortex in adolescents during cognitive control tasks: a functional near-infrared spectroscopy study

**DOI:** 10.1117/1.NPh.13.S1.S13009

**Published:** 2026-02-18

**Authors:** Yuna Hong, Hyunchan Hwang, Young-Don Son, Gangta Choi, Hye Ri Kim, Doug Hyun Han

**Affiliations:** a Chung-Ang University, College of Medicine, Department of Psychiatry, Seoul, Republic of Korea; b Gachon University, Department of Biomedical Engineering, Seongnam, Republic of Korea

**Keywords:** attention deficit hyperactivity disorder, functional near-infrared spectroscopy, dorsolateral prefrontal cortex, adolescent

## Abstract

**Significance:**

Adolescents with attention-deficit/hyperactivity disorder (ADHD) have reduced dorsolateral prefrontal cortex (DLPFC) activation during cognitive control tasks and weaker attention performance, with higher DLPFC activity linked to better complex attention, showing a connection between neural function and behavior. These findings support prefrontal dysfunction and hemispheric asymmetry in ADHD adolescents.

**Aim:**

We aimed to compare brain activation during cognitive control tasks between adolescents with ADHD and typically developing (TD) individuals using functional near-infrared spectroscopy (fNIRS).

**Approach**

Sixty-eight adolescents participated: 30 ADHD and 38 TD, and were assessed using the Korean ADHD Rating Scale, Comprehensive Attention Test, Korean Beck Depression Inventory-II, and Korean Beck Anxiety Inventory. Brain activation during the Stroop Color and Word Test (ST) was measured with fNIRS. Psychological scales, cognition-related tests, and brain activation data were analyzed.

**Results:**

The ADHD group recorded decreased scores for congruence and incongruence of ST compared with the TD group. TD adolescents showed significantly greater increases in oxygenated hemoglobin concentration ($\Delta {\mathrm{HbO}}_{2}$) in the prefrontal cortex (DLPFC) than adolescents with ADHD. DLPFC activation was negatively correlated with ADHD symptom severity (all participants). Correlations were observed between DLPFC $\Delta {\mathrm{HbO}}_{2}$ and cognitive function, particularly complex attention.

**Conclusions:**

We found distinct brain activation patterns in the DLPFC for ADHD adolescents during cognitive control tasks. fNIRS measured these differences, helping to understand the developmental trajectory of ADHD in adolescents and providing evidence of functional impairment in complex executive function tasks.

## 1 Introduction

Attention-deficit/hyperactivity disorder (ADHD) is a prevalent neurodevelopmental disorder observed in childhood and can be classified into inattentive, impulsive, and combined subtypes. Its global prevalence is ∼7.6% and 6.76% in children and adults, respectively.^1^^,^^2^ The primary symptoms of ADHD include inattention, impulsivity, and hyperactivity, with problematic behaviors typically manifesting and diagnosed during early childhood, particularly when academic and interpersonal relationships become more important. Early diagnosis and intervention during this critical developmental period significantly influence the management of symptoms in later life. Consequently, most studies have focused on patients aged 6 to 12 years. However, ADHD should not be regarded solely as a childhood issue but rather as a lifelong disorder that requires a comprehensive understanding and exploration of its developmental trajectory from childhood to adulthood.^3^ Therefore, there has been a growing effort to examine and understand the developmental trajectory of ADHD across the entire lifespan.^4^^,^^5^ However, research focusing on adolescents remains relatively scarce.^6^^,^^7^

One of the most widely accepted theories explaining core deficits in ADHD is the executive function deficit theory,^8^ which states that ADHD is not only characterized by a lack of attention or excessive hyperactivity, but is also fundamentally linked to deficits in various executive functions. These executive functions encompass a broad range of cognitive processes, including planning and organization, working memory, inhibitory control, and cognitive flexibility. Individuals with ADHD exhibit impairments in simple attention (i.e., the ability to focus on a specific stimulus), complex attention (i.e., the ability to allocate and sustain attention across multiple stimuli), and working memory (i.e., the ability to temporarily retain and effectively manipulate information). Such deficits can result in significant challenges for adolescents with ADHD, affecting both academic performance and social interactions.^9^

These difficulties often persist into adulthood. Longitudinal research suggests that ∼50% of children diagnosed with ADHD continue to show symptoms later in life, which can lead to a wide range of difficulties in adapting to adulthood.^10^^,^^11^ Therefore, ADHD is understood as a complex neurodevelopmental disorder that continuously influences an individual’s developmental trajectory and overall function throughout their lifespan. As symptoms observed during adolescence can adversely affect adulthood, timely and targeted interventions during this critical period are essential.

Despite decades of active research, the precise causes of ADHD have yet to be definitively identified. Current understanding suggests that ADHD arises from a complex interplay of genetic, neurobiological, and environmental factors. From a neurobiological perspective, research has focused on the structural and functional differences in the brain. Studies have reported associations between ADHD and brain regions such as the prefrontal lobe, basal ganglia, and gray matter, as well as cortical thickness and features of the limbic system.^12^^–^^15^ In particular, ADHD is closely linked to deficits in information processing within the prefrontal cortex, which contribute to impairments in executive functions such as cognitive control.^16^^–^^19^

Neuroimaging techniques, such as electroencephalography (EEG)^20^^,^^21^ and functional magnetic resonance imaging (fMRI),^22^ have been employed to explore the neural mechanisms underlying ADHD. Conventional neuroimaging techniques have several advantages. fMRI, which measures and visualizes the activation of specific brain regions by detecting changes in cerebral blood flow, is used to analyze the connectivity among various brain areas and is known for its high spatial resolution.^23^ However, fMRI has several limitations, including high cost, restricted equipment mobility, and significant noise levels.^24^ EEG is more versatile in its usage, with better temporal resolution^25^ and, generally speaking, more cost-efficient than fMRI. Due to these advantages, its potential use as a biomarker for ADHD has been extensively researched.^26^ However, EEGs have some drawbacks, including low spatial resolution and sensitivity to movement and environmental artifacts,^27^ although technical improvements have been made to combat these in the evaluation of ADHD.^28^

Functional near-infrared spectroscopy (fNIRS) has emerged as a promising alternative to better understand the neurobiological foundations of ADHD.^29^ fNIRS differs from conventional neuroimaging techniques in that it has a higher temporal resolution than fMRI and better spatial resolution than EEG when limited to the prefrontal lobe.^30^ It is also more tolerant to motion artifacts, lighter, and more flexible,^31^ making it well-suited for assessing brain activity in young participants without causing significant discomfort or sedation. Even though both fNIRS (optode shifts)^32^ and EEG (muscle artifacts)^33^ are susceptible to motion-related noise, children with ADHD, who often struggle to remain still for extended periods, may benefit from the adaptability of fNIRS because it has less noise due to movement than EEG or fMRI.^33^^,^^34^ Thus, fNIRS serves as a viable alternative for evaluating prefrontal lobe function in younger individuals.^35^ In addition, it is less dependent on environmental noise^30^ and easier to apply. Therefore, it does not require as much technical expertise as EEG or fMRI technicians to use.^36^ These factors contribute to the cost-effectiveness of fNIRS, making it a viable alternative method for brain evaluation when MRI or EEG are unavailable.

Calub et al.^37^ used fNIRS to measure prefrontal lobe activity while performing a visual array task and revealed significant deficits in attention regulation among children with ADHD compared with their typically developing (TD) peers. Although the study provides valuable foundational data demonstrating differences in attention control between the ADHD and control groups using fNIRS, its applicability is limited by the age range of the participants, which was restricted to children aged 8 to 12 years. Many studies worldwide have examined children using fNIRS,^37^^–^^39^ but research specifically assessing adolescent ADHD populations using this method remains limited. This study aimed to address the limitations of previous studies focusing on childhood by examining adolescents aged 13 to 18. Also, ADHD is associated with a high rate of comorbidity with various other psychiatric disorders.^40^^,^^41^ By examining pure ADHD cases without comorbid conditions, this study aimed to provide a more comprehensive analysis of attention regulation and prefrontal lobe activation in adolescent populations.

Accordingly, we hypothesized that adolescents with ADHD and TD adolescents differ in prefrontal lobe function, as measured by fNIRS, and that adolescents with ADHD experience more difficulty performing tasks that require attention (such as the Stroop Color and Word Test) than do TD adolescents.

## 2 Materials and Methods

### 2.1 Participants and Procedures

To recruit participants for this study, posters were distributed at the psychiatry outpatient department of Chung-Ang University Hospital and on internal bulletin boards at Wee Centers operated by the Seoul Metropolitan Office of Education and nearby schools. Participant recruitment was conducted from October 2024 to November 2024. The inclusion criteria for the ADHD group were as follows: (1) adolescents aged 13 to 18 years, (2) diagnosed with ADHD based on the DSM-5 criteria, (3) no diagnosis of any psychiatric disorder other than ADHD within 12 months prior to study participation, and (4) no history of developmental disorders or neurological conditions. The inclusion criteria for the TD group were as follows: (1) adolescents aged 13 to 18 years, (2) no psychiatric diagnosis within the past 12 months, and (3) no history of developmental disorders or neurological conditions. The exclusion criteria for both groups were as follows: (1) those who did not consent to participate, (2) individuals with severe medical conditions requiring hospitalization, (3) individuals with skin lesions or tattoos on the forehead that would interfere with fNIRS imaging, and (4) participants deemed unsuitable for the study by the investigator for any other reason.

Participants who were interested in the study would contact the researchers and were given more detailed information on the study. If they agreed to participate in the study, the participant and their legal guardian would give written consent. After that, the researchers would check for the exclusion and inclusion criteria. The participants would also meet with a child and adolescent psychiatrist for evaluation of their diagnosis and any other comorbid disorders.

Initially, 32 patients participated in this study. One adolescent was excluded because of severe depression, and another was excluded because of severe anxiety. In the control group of 38 adolescents, no participants were excluded from the study. The participants and their legal guardians provided written informed consent after the study procedures and goals were explained in detail. All ADHD participants recruited had the combined presentation to ensure group homogeneity.

The fNIRS measurements were conducted for all participants between 10:00 a.m. and 5:00 p.m. in an independent air-conditioned laboratory shielded from external light. All participants were taking stimulants (methylphenidate) for ADHD and therefore were ordered to discontinue their medication for five days prior to the fNIRS session and were instructed to avoid strenuous physical activity on the day of measurement. The room temperature was maintained at 23°C to prevent excessive perspiration and enhance participant comfort, thereby minimizing motion-induced signal artifacts. This temperature is widely used as an appropriate environmental condition for fNIRS measurements because it allows the collection of data under stable physiological states.^42^ During the recordings, the participants remained comfortably seated and were instructed to remain as still as possible. Prior guidance was provided to minimize excessive movement and blinking.

During the Stroop Task (ST), the prefrontal cortex activation was measured using fNIRS. Baseline activation was recorded during a 3-min resting period prior to task initiation. Following the baseline measurement, a 10-s preparation period was provided. The participants then completed a computerized version of the ST for 7 min, during which the prefrontal cortex activity was continuously recorded using fNIRS.

After fNIRS measurements, the participants completed demographic assessments, including age, grade level, and sex, as well as psychological assessments using the Korean Beck Depression Inventory-II (K-BDI-II), Korean Beck Anxiety Inventory (K-BAI), Korean ADHD Rating Scale (K-ARS), and Comprehensive Attention Test (CAT).

This study was approved by the Institutional Review Board (IRB) of Chung-Ang University Hospital (IRB No. 2409-012-615), and all procedures were conducted in accordance with the Declaration of Helsinki. Written informed consent was obtained from all the participants and their legal guardians.

### 2.2 Assessment Instruments

#### 2.2.1 Korean Attention Deficit Hyperactivity Disorder Rating Scale

The K-ARS was developed to evaluate ADHD symptoms in school-aged children.^43^ It has been translated into and validated for use in Korean.^44^ Its structure is based on the DSM-IV diagnostic criteria and contains 18 items. Each item is rated on a scale from 0 to 3, where 0 = “not at all,” 1 = “sometimes,” 2 = “often,” and 3 = “very often.” The total score of the odd-numbered items was designed to measure symptoms of inattention, whereas the total score of the even-numbered items assessed symptoms of hyperactivity–impulsivity.

#### 2.2.2 Korean Beck Depression Inventory-II

The K-BDI-II is a self-report questionnaire used to evaluate depression in adolescents and adults.^45^ It comprises 21 items, each rated on a 4-point Likert scale ranging from 0 to 3, based on how well the statement describes the participant’s mood. The total score ranged from 0 to 63, with higher scores indicating greater severity of depression.

#### 2.2.3 Korean Beck Anxiety Inventory

The K-BAI is a self-report questionnaire used to measure anxiety in adolescents and adults.^46^ It comprises 21 items covering the cognitive, emotional, and physical aspects of anxiety. Participants rated the severity of symptoms experienced over the past week on a 4-point Likert scale ranging from 0 to 3. The total score ranges from 0 to 63, with higher scores indicating higher anxiety levels.

#### 2.2.4 Comprehensive Attention Test

The CAT is a computerized continuous performance task designed to comprehensively measure various types of attention in individuals aged 4 to 49 years. The Korean version was developed and standardized in 2009 and includes six test components: selective visual attention, selective auditory attention, inhibitory attention, interference attention, divided attention, and working memory.^47^ The test can be administered by combining individual subtests based on the participant’s age or situation or by administering the whole subtest. All six subtests were used in this study; completing them took ∼40  min. The selective visual attention task measures the ability to respond quickly to a target stimulus by having participants identify a specific stimulus among several visual stimuli. The selective auditory attention task assesses the ability to respond to auditory cues by requiring participants to react as quickly as possible when they hear a specific sound from among various auditory stimuli. The inhibitory attention task measures the ability to maintain attention and refrain from impulsivity by requiring participants to respond consistently to stimuli, except for one target stimulus. The interference attention task measures the ability to ignore distracting stimuli, focuses on the target stimulus, and involves selecting arrows corresponding to the directions in which the centers of the five boxes open. The divided attention task, which evaluates the ability to process multiple stimuli simultaneously, presents visual and auditory stimuli simultaneously and requires participants to react as quickly as possible when a previously shown image or sound is repeated. The working memory task includes both forward and backward tasks. The former requires participants to remember and click stimuli in the order presented, whereas the latter requires remembering and clicking the stimuli in the reverse order, assessing the ability to recall and manage a sequence of stimuli. Evaluating each subtest allowed for a multifaceted analysis of the participants’ cognitive functions.

#### 2.2.5 Stroop Color and Word Test

The ST was developed to evaluate the ability of conflict processing and inhibition, which is managed by the prefrontal lobe.^48^ Three ST parameters were evaluated. Word (Stroop word) and color (Stroop color) readings were performed in the congruent condition, and color word reading (Stroop color–word) was performed in the incongruent condition. Stroop words represent color words written in black. For instance, words such as “green” or “blue” are displayed in black. Stroop colors are presented in a congruent condition so that the meaning of the word and the color of the letters match. For example, the word “green” is presented in green ink. In this condition, there was no discrepancy between the word meaning and ink color (congruent), allowing participants to easily recognize and respond. Three colors, green, blue, and red, were used. The Stroop color–word was performed as an incongruent condition so that the meaning of the word and the color of the letters did not match. For example, the word “green” might be written in blue ink. In this scenario, interference occurred in the participant’s cognitive process, as there was a conflict between the meaning of the word and the ink color. Participants had to ignore the meaning of the word and focus on the color, thus demanding greater cognitive effort.

The Stroop task was administered via tablet PC (Galaxy Book S9+) and was based on the Korean version of the Stroop Color and Word Test,^49^ which was made by modifying the English version.^50^ One hundred words per test (word, color, and word–color) were provided in 5 rows with 20 words in each row. The test administrator would first give the participant a verbal explanation of the testing procedures, with a five-word example they could use to check if the participant had understood the procedure. Participants were asked to read the word or color loudly, and if they happened to be wrong, the test administrator would tell them that it is wrong, and they would read it again. When the participant had fully understood the test and was ready, the test would begin with 45 s per test. After 45 s, the participant would mark the final word they read before the 45-s mark. Participants had a 10-s time period before each test, and a blank screen was provided for those time periods.

#### 2.2.6 Functional near-infrared spectroscopy

In this study, the brain activity was assessed using fNIRS. When a specific brain region becomes active, the associated increase in metabolic demand leads to elevated cerebral blood flow, increasing oxygenated hemoglobin and a relative decrease in deoxygenated hemoglobin. The fNIRS device operates in continuous wave mode, emitting constant-intensity near-infrared light that penetrates the skull.

**Fig. 1:**
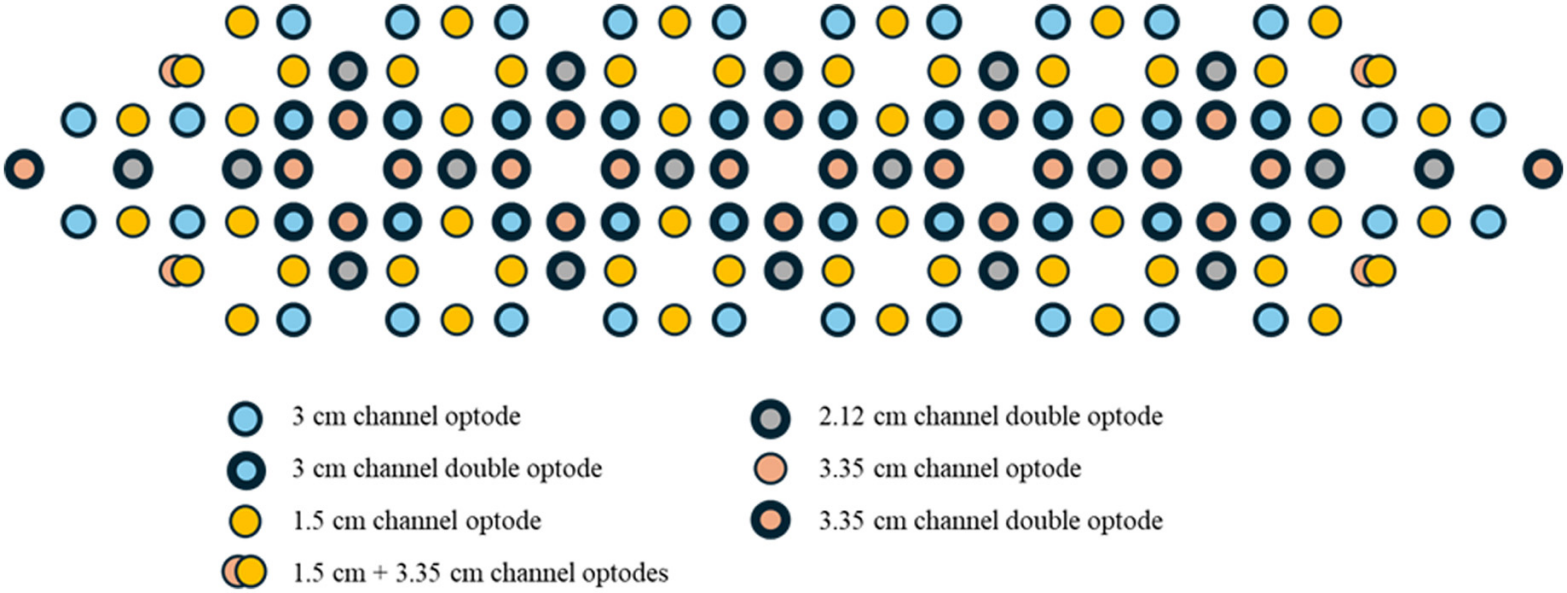
Visualization of optode placement.

The fNIRS device used in our study was the NIRSIT device (OBELAB, Inc., Seoul, Republic of Korea). This device has specialized detectors that measure the reflected light to quantify changes in oxygenated and deoxygenated hemoglobin concentrations. The change in oxygenated hemoglobin concentration in a specific brain region can be used as the primary quantitative metric representing brain activation. The device features 24 laser diodes (sources) emitting at dual wavelengths (780 and 850 nm) and 32 photodetectors with analog signals digitized through a 16-bit analog-to-digital (A/D) conversion for subsequent processing, achieving a sampling rate of 8.138 Hz. The sources and detectors were spaced 1.5 cm apart; both 1.5- and 3-cm channels were used during preprocessing and artifact correction. The 1.5-cm channels were utilized to correct for motion artifacts and noise in the 3-cm channels. Following the software and analysis protocol provided by the fNIRS device manufacturer, the 204-channel data including 1.5-cm channels were integrated into 48 channel groups using a distance-based interpolation algorithm. Subsequently, final statistical analyses focused on 3-cm channels, as this separation distance provides optimal cortical penetration depth for measuring prefrontal hemodynamic responses. This setup produced 48 measurement channels, which were calibrated for noise reduction and spatially interpolated to eight bilateral prefrontal ROIs (Fig. 1).

Light signals at each wavelength were subjected to a band-pass filter (0.005 to 0.1 Hz) to minimize interference from physiological motion noise, such as cardiac pulsation (∼1  Hz) and respiration (0.2 to 0.3 Hz). Channels with a signal-to-noise ratio below 30 dB were excluded to maintain accurate hemodynamic analysis. For spatial continuity, the signals from the rejected channels were reconstructed via channel-wise interpolation using neighboring valid channels. Motion artifacts were corrected using the built-in accelerometer and gyroscope data. Head angle features were regressed onto the raw intensity signal, and fitted components with a correlation above 0.6 were removed to reduce motion-related noise. In addition, temporal segments were continuously evaluated for motion artifacts based on accelerometer and gyroscope data. Segments where motion parameters (acceleration magnitude and angular velocity) exceeded the manufacturer’s predefined thresholds (acceleration, $1.8\;m/s^{2}$; angular velocity, 1.8  deg/s) or where the optical signal exhibited abrupt intensity changes beyond acceptable ranges (ΔOD 0.005 per 10 time points) were automatically flagged as artifacts.^51^ These contaminated segments were removed from the time series, and the resulting gaps were reconstructed using temporal interpolation from surrounding clean signal periods, following the manufacturer’s standardized protocol (Table 1).

**Table 1:** Rejected channels and signals.

Group	Number of rejected channels (<30 dB)			Motion rejection (%)
	780 nm	850 nm	Total	
TD group	23.8 ± 12.6	21.8 ± 13.9	36.9 ± 16.0	8.8 ± 2.0
ADHD group	29.9 ± 11.7	23.3 ± 13.3	39.2 ± 14.5	11.1 ± 2.4

The relative hemodynamic changes for each channel during resting and ST were calculated using the modified Beer–Lambert law,^52^ which models hemoglobin concentration change (Δc) as being directly proportional to the change in optical density and inversely proportional to the product of the molar extinction coefficient (ε), differential path length factor (DPF), and source–detector distance (L); that is, Δc∝ΔOD/(ε×DPF×L). Based on this method, brain activation was quantified using the mean oxygenated hemoglobin concentration during the resting and task states.

**Fig. 2:**
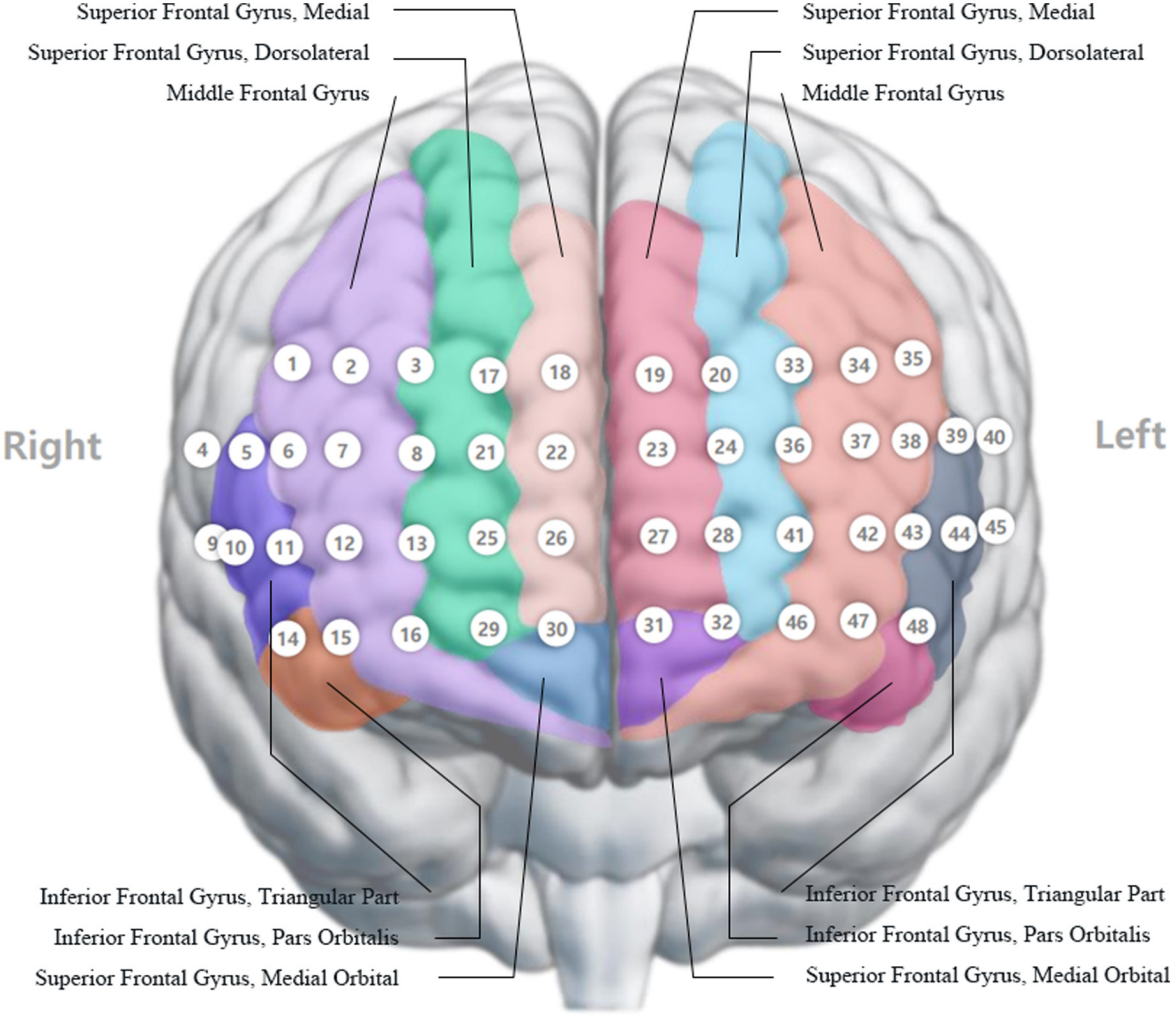
Visual representation of the fNIRS device and its placement of measurement channels in relation to the brain. Adapted with permission of OBELAB, Inc. [OBELAB, Inc. (2022)]. NIRSIT Channel Information, Seoul, Republic of Korea.^53^

Hemodynamic data processing involved sequential baseline correction steps. First, during the conversion from raw light intensity to optical density, signals were referenced to an intensity baseline at 15 s prior to stimulus onset (−15  s), establishing a stable pre-task reference point. These optical density values were subsequently transformed into hemoglobin concentration changes via the modified Beer–Lambert law. Following concentration estimation, each trial underwent trial-specific baseline correction by subtracting the mean oxygenated hemoglobin concentration calculated over the −2- to 0-s window immediately preceding stimulus onset. Individual results from multiple trials were block-averaged and aggregated into a grand average. The accumulated oxygenated hemoglobin (${\mathrm{accHbO}}_{2}$) values computed for these stimulation periods indicate activation of the prefrontal cortex. The difference between the ${\mathrm{accHbO}}_{2}$ of resting state fNIRS and the ${\mathrm{accHbO}}_{2}$ of fNIRS during the ST was defined as the mean change of accumulated oxygenated hemoglobin ($\Delta {\mathrm{HbO}}_{2}$). The means and standard deviations of the ${\mathrm{accHbO}}_{2}$ were calculated for the defined regions of interest within the right and left dorsolateral prefrontal cortex (DLPFC), ventrolateral prefrontal cortex (VLPFC), frontopolar cortex (FPC), and orbitofrontal cortex (OFC). Specifically, the right and left DLPFC were represented by channels 1, 2, 3, 5, 6, 11, 17, and 18 and channels 19, 20, 33, 34, 35, 38, 39, and 43, respectively. The right and left VLPFC were represented by channels 4, 9, and 10 and channels 40, 44, and 45, respectively. The right and left FPC were represented by channels 7, 8, 12, 13, 21, 22, 25, and 26 and channels 23, 24, 27, 28, 36, 37, 41, and 42, respectively. The right and left OFC were represented by channels 14, 15, 16, 29, and 30 and channels 31, 32, 46, 47, and 48, respectively (Fig. 2).

### 2.3 Statistical Analysis

SPSS 25 (SPSS Inc., Chicago, Illinois, United States) was used for statistical analysis of the data. Differences in demographic variables were analyzed using independent t-tests to compare the mean values among the groups. For categorical variables such as socioeconomic status, the chi-square test was used to assess the differences among groups. Independent t-tests were also employed to analyze the K-BDI-II, K-BAI, and K-ARS scores collected from adolescents in the ADHD and TD groups. Pearson’s correlation analysis was conducted to examine the relationship among the CAT scores, K-ARS scores, and $\Delta {\mathrm{HbO}}_{2}$. To account for multiple analyses of the various subtypes of cognitive tests between the ADHD and TD groups, a Bonferroni correction was performed, and the significance level for the 12 comparisons of attention and working memory test scores between the ADHD and TD groups was adjusted accordingly. A significant p-value was set at 0.05/12=0.004. In all other cases, a p-value of 0.05 was considered statistically significant.

*Post hoc* power analysis was conducted to determine the achieved statistical power for the study’s primary finding: the difference in $\Delta {\mathrm{HbO}}_{2}$ in the DLPFC between the ADHD and TD groups. The left DLPFC was chosen over the right DLPFC because it was more statistically sound. The observed effect size (Cohen’s d) of $\Delta {\mathrm{HbO}}_{2}$ in the right DLPFC was calculated to be 0.74, which is considered a medium-to-large effect. Based on this effect size and the study’s total sample of 68 participants (30 ADHD and 38 TD), the achieved power was 0.84. As this result is above the conventional 0.80 threshold, we decided that the finding is statistically robust and not likely due to a type II error.

## 3 Results

### 3.1 Comparison of Demographic and Psychological Characteristics Between Adolescents with ADHD and TD Adolescents

There were no significant differences in demographic factors, including age, sex, and socioeconomic status, between adolescents with ADHD and TD adolescents.

There were no significant differences in the K-BDI-II and K-BAI scores between adolescents with ADHD and TD adolescents. However, the K-ARS scores of adolescents with ADHD were significantly higher than those of the TD adolescents (Table 2).

**Table 2:** Comparison of demographic and psychological characteristics.

	TD (N=38)	ADHD (N=30)	Statistics
Age	14.58 ± 1.31	14.10 ± 1.32	t=−1.49, p=0.14
Sex	—	—	$\chi^{2}=0.60$, p=0.62
Male (%)	23 (60.5%)	20 (66.7%)	—
Female (%)	15 (39.5%)	10 (33.3%)	—
Socioeconomic status	—	—	$\chi^{2}=0.39$, p=0.82
Low (%)	3 (7.9%)	2 (6.7%)	—
Middle (%)	28 (73.7%)	24 (80.0%)	—
High (%)	7 (18.4%)	4 (13.3%)	—
K-BDI	11.61 ± 6.77	13.60 ± 5.65	t=1.30, p=0.20
K-BAI	6.87 ± 7.06	7.80 ± 6.75	t=0.55, p=0.58
K-ARS*	3.24 ± 2.83	15.77 ± 6.31	t=10.10, p<0.001

K-BDI, Korean Beck Depression Inventory-II; K-BAI, Korean Beck Anxiety Inventory; K-ARS, Korean Attention Deficit Hyperactivity Disorder Rating Scale.

* p<0.05, M ± SD: mean ± standard deviation.

### 3.2 Comparison of Attention and Working Memory Assessment Tests Between Adolescents with ADHD and TD Adolescents

In the CAT, adolescents with ADHD showed more errors and lower scores in all of the subscales, with significantly higher errors in interference commission and divided omission when compared with those observed in TD adolescents (Table 2).

In the ST, adolescents with ADHD showed decreased t-scores for the color–word test and interference scores (difference among incongruent–congruent trials) compared with TD adolescents (Table 3).

**Table 3:** Comparison of attention and working memory assessment tests between adolescents with ADHD and TD adolescents.

	TD (N=38)	ADHD (N=30)	Statistics
Comprehensive attention test (number of errors, unless otherwise stated)			
Selective visual omission^a^	5.32 ± 17.11	6.27 ± 18.80	t=0.21, p=0.83
Selective visual commission^a^	2.16 ± 3.53	3.63 ± 5.32	t=1.36, p=0.18
Selective auditory omission^a^	2.14 ± 3.07	3.73 ± 4.80	t=1.65, p=0.10
Selective auditory commission	2.47 ± 2.98	4.47 ± 4.90	t=2.07, p=0.04
Inhibition omission	4.63 ± 6.64	13.90 ± 16.99	t=2.82, p=0.008
Inhibition commission	10.05 ± 8.68	18.20 ± 15.13	t=2.79, p=0.007
Interference omission	2.50 ± 2.76	10.30 ± 13.88	t=3.03, p=0.005
Interference commission^*^	5.63 ± 4.97	14.77 ± 14.18	t=3.37, p=0.002
Divided omission^*^	5.16 ± 6.03	14.23 ± 9.86	t=4.43, p<0.001
Divided commission	5.00 ± 3.46	10.50 ± 9.98	t=2.89, p=0.007
Working memory forward (scores)	6.02 ± 1.08	5.23 ± 1.91	t=−2.17, p=0.03
Working memory backward (scores)	6.13 ± 0.70	5.20 ± 1.83	t=−2.64, p=0.01
Stroop Color and Word Test^b^ (t-scores)			
Word	50.11 ± 10.16	45.50 ± 13.75	t=−1.57, p=0.12
Color	53.92 ± 11.72	49.97 ± 11.59	t=−1.37, p=0.18
Word–color^*^	50.94 ± 12.97	41.93 ± 8.95	t=−3.22, p=0.002
Interference^*^	51.53 ± 12.48	43.73 ± 7.67	t=−2.98, p=0.004

* p<0.004 was considered significant as result of the Bonferroni correction M ± SD: mean ± standard deviation.

M ± SD: mean ± standard deviation.

a One set of data from the typically developing group was missing.

b Two sets of data from the typically developing group were missing.

### 3.3 Comparison of $\Delta {\mathrm{HbO}}_{2}$ Within the Brain in Response to the Stroop Test

In response to the ST, the $\Delta {\mathrm{HbO}}_{2}$ within the left (t=3.03, p=0.003) and right (t=2.02, p=0.047) DLPFC in TD adolescents significantly increased compared with that in adolescents with ADHD.

**Fig. 3:**
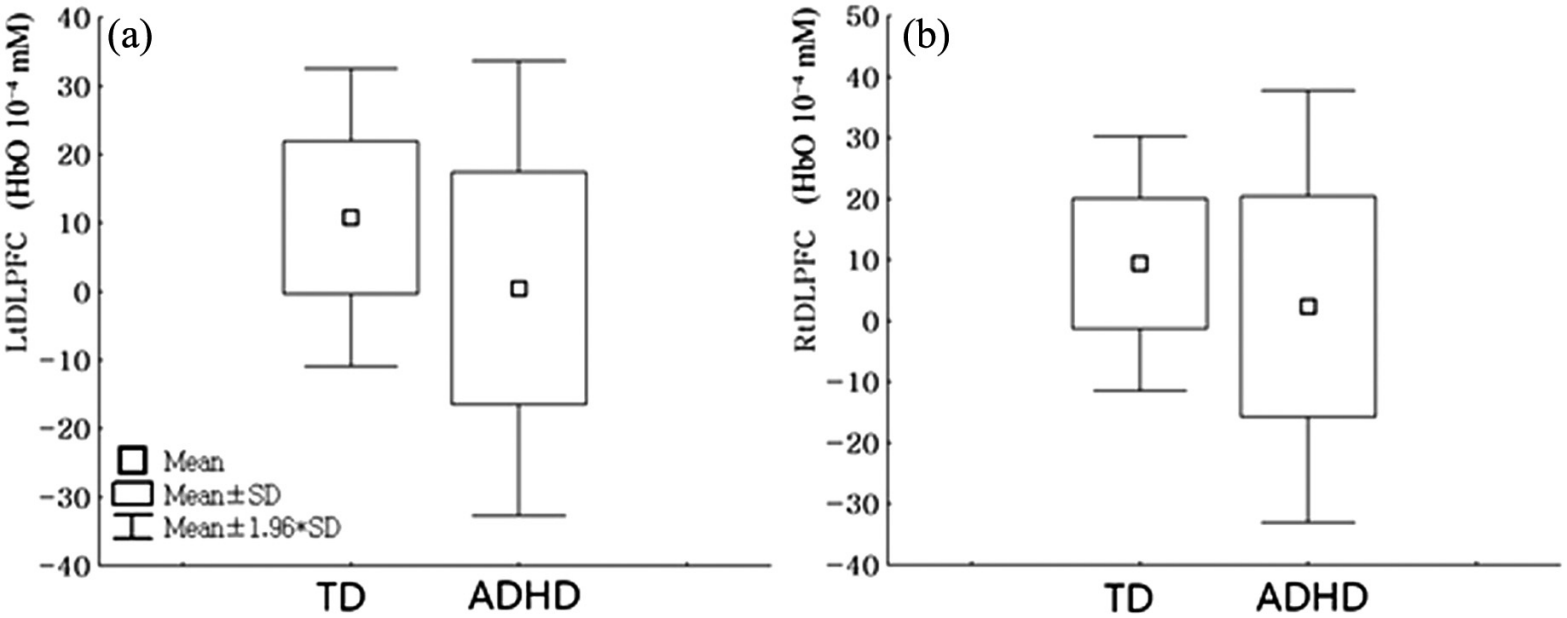
Comparison of oxygen saturation changes in response to the Stroop Test between adolescents with ADHD and TD adolescents. LDLPFC, left dorsolateral prefrontal cortex; RDLPFC, right dorsolateral prefrontal cortex; SD, standard deviation; TD, typically developing adolescent group; ADHD, attention deficit/hyperactivity disorder adolescent group; $\Delta {\mathrm{HbO}}_{2}$, delta value of accumulated oxygenated hemoglobin. (a) Comparison of oxygen saturation changes within left DLPFC in response to the Stroop Test between adolescents with ADHD and TD adolescents, TD: 10.79±11.11, ADHD: 0.45±16.94, t=3.03, p=0.003. (b) Comparison of oxygen saturation changes within right DLPFC in response to the Stroop Test between adolescents with ADHD and TD adolescents, TD: 9.41±10.65, ADHD: 2.32±17.08, t=2.02, p=0.047.

There were no differences in $\Delta {\mathrm{HbO}}_{2}$ changes within other brain areas between adolescents with ADHD and TD adolescents (Fig. 3).

### 3.4 Correlations Between K-ARS Scores and $\Delta {\mathrm{HbO}}_{2}$ in Response to the Stroop Test

The K-ARS scores were negatively correlated with $\Delta {\mathrm{HbO}}_{2}$ within the left (r=−0.45, p<0.01) and right (r=−0.53, p<0.01) DLPFC in all participants.

In the sub-group analysis, the K-ARS scores were negatively correlated with $\Delta {\mathrm{HbO}}_{2}$ within the right DLPFC (r=−0.73, p<0.01) in adolescents with ADHD. However, the K-ARS scores showed no significant correlation with $\Delta {\mathrm{HbO}}_{2}$ within the left DLPFC (r=−0.33, p=0.07).

**Fig. 4:**
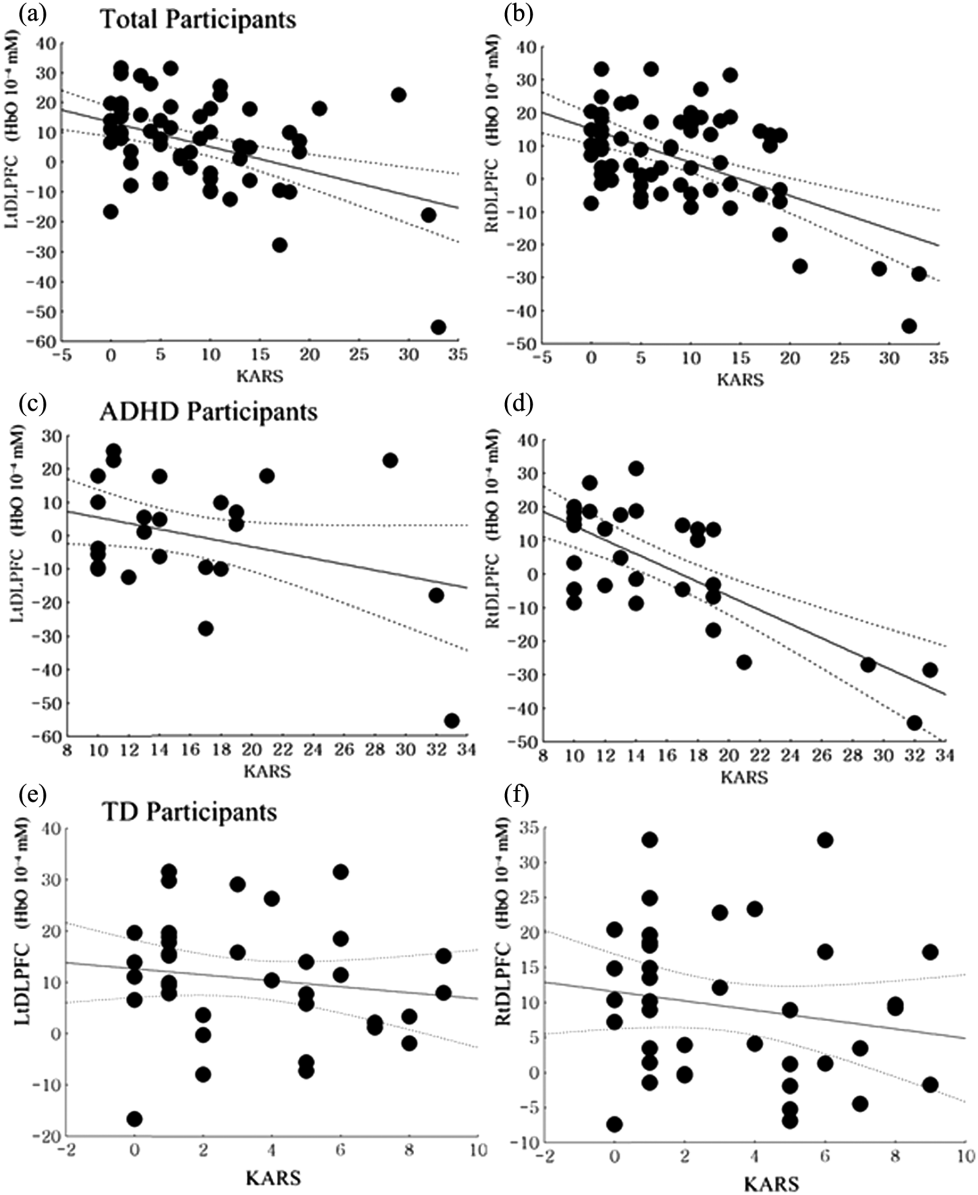
Correlations between K-ARS scores and brain activity in response to the Stroop Test. (a) Correlation between the Korean Attention Deficit Hyperactivity Disorder Rating Scale (K-ARS) and the delta value of accumulated oxygenated hemoglobin ($\Delta {\mathrm{HbO}}_{2}$) within the left DLPFC in all participants (r=−0.45, p<0.01). (b) Correlation between the K-ARS scores and $\Delta {\mathrm{HbO}}_{2}$ within the right DLPFC in all participants (r=−0.53, p<0.01). (c) Correlation between the K-ARS scores and $\Delta {\mathrm{HbO}}_{2}$ within the left DLPFC in the ADHD group (r=−0.33, p=0.07). (d) Correlation between the K-ARS scores and $\Delta {\mathrm{HbO}}_{2}$ within the right DLPFC in the ADHD group (r=−0.73, p<0.01). (e) Correlation between the K-ARS scores and $\Delta {\mathrm{HbO}}_{2}$ within the left DLPFC in the TD group (r=−0.15, p=0.37). (f) Correlation between the K-ARS scores and $\Delta {\mathrm{HbO}}_{2}$ within the right DLPFC in the TD group (r=−0.18, p=0.29).

In TD adolescents, there were no significant correlations between K-ARS scores and $\Delta {\mathrm{HbO}}_{2}$ within the left (r=−0.15, p=0.37) and right (r=−0.18, p=0.29) DLPFC (Fig. 4).

### 3.5 Correlations Between the CAT Test and $\Delta {\mathrm{HbO}}_{2}$ in Response to the Stroop Test

The $\Delta {\mathrm{HbO}}_{2}$ within the right DLPFC was negatively correlated with interference omission (r=−0.73, p<0.001), interference commission (r=−0.55, p<0.001), divided omission (r=−0.55, p<0.001), and divided commission (r=−0.68, p<0.001) but positively correlated with working memory backward (r=0.34, p=0.005) in all participants.

The $\Delta {\mathrm{HbO}}_{2}$ within the left DLPFC was negatively correlated with interference omission (r=−0.315, p=0.009) and divided commission (r=−0.311, p=0.010) in all participants.

In adolescents with ADHD, the $\Delta {\mathrm{HbO}}_{2}$ within the right DLPFC was negatively correlated with interference omission (r=−0.85, p<0.001), interference commission (r=−0.72, p<0.001), divided omission (r=−0.68, p<0.001), and divided commission (r=−0.78, p<0.001). In adolescents with ADHD, there were no significant correlations between $\Delta {\mathrm{HbO}}_{2}$ levels within the left DLPFC and CAT subcategories.

## 4 Discussion

In the current study, adolescents with ADHD showed decreased t-scores for the word color test and interference of the Stroop task compared with TD adolescents. In brain activity, adolescents with ADHD did not exhibit significant changes in the difference of oxygenated hemoglobin between resting and task performance in the DLPFC during task performance, whereas the TD group demonstrated significant increases in $\Delta {\mathrm{HbO}}_{2}$. In addition, a negative correlation was found between DLPFC oxygen saturation and clinical characteristics as measured by the K-ARS. Correlations were also observed between DLPFC $\Delta {\mathrm{HbO}}_{2}$ and cognitive function measured by the CAT, particularly in areas related to complex attention.

A comparison of attention and cognitive function between adolescents with ADHD and TD adolescents revealed no significant differences in simple selective attention tasks in the CAT or in congruent conditions of the ST. However, in more complex attention tasks, such as those involving inhibition, interference, and divided attention, as well as in the incongruent ST, where interference effects occurred, the ADHD group demonstrated a tendency toward increased errors and lower working memory scores, suggesting that adolescents with ADHD struggle with tasks that require higher-level cognitive function. Previous research comparing executive function between ADHD and TD groups similarly reported that individuals with ADHD face more difficulty in tasks that demand complex cognitive processes.^17^^,^^54^ In addition, in the word–color task of the ST, where the meaning of color and word conflict, a higher level of attention and executive function is required, and the ADHD group appears to experience greater difficulty than the TD group because of their reduced ability to process interfering stimuli.^55^ The results of the present study are consistent with these findings, suggesting that these difficulties may be linked to functional changes in the brain. ADHD has been reported to be closely associated with deficits in information processing within the prefrontal cortex, particularly in the DLPFC.^12^^–^^15^ The DLPFC plays a crucial role in high-level cognitive functions (e.g., sustained attention, working memory, and inhibitory control), and dysfunction in this region can result in impairments in executive function.^56^ Functional and structural changes in the DLPFC may influence the severity and manifestation of ADHD symptoms throughout development,^57^ potentially exacerbating cognitive difficulties with complex tasks in adolescents with ADHD. Consequently, functional deficits in the DLPFC may be an underlying reason why adolescents with ADHD experience a greater cognitive burden in high-demand cognitive tasks.

The comparison of $\Delta {\mathrm{HbO}}_{2}$ in the brain during ST revealed that adolescents in the ADHD group did not show significant increases in $\Delta {\mathrm{HbO}}_{2}$ in the left and right DLPFC, whereas TD adolescents exhibited marked activation. This suggests that the DLPFC, the brain region responsible for executive function, does not achieve normal activation in adolescents with ADHD. A recent fNIRS review article by Gossé et al.^58^ reported consistent hypoactivity in the prefrontal cortex in multiple studies using the Stroop paradigm with children and adolescents aged 6 to 16 years with ADHD. These outcomes have been consistently observed in previous studies.^59^ The present study further clarified the differences in DLPFC activation among adolescents aged 13 to 18 years with ADHD and their TD peers to provide foundational data that could contribute to a more precise understanding of the role of the DLPFC in adolescent ADHD.

Analyzing the correlation between the K-ARS scores and $\Delta {\mathrm{HbO}}_{2}$ to compare the differences between the left and right DLPFC revealed a negative correlation between ADHD severity and DLPFC activation changes across all groups. However, in the ADHD group, this negative correlation was maintained only in the right DLPFC, and no significant correlation was observed in the left DLPFC. Although previous studies have shown relatively consistent results regarding the right DLPFC, findings related to the left DLPFC are more variable, leading to differing interpretations of the roles of the left and right DLPFC in ADHD. For instance, Wu et al.^60^ used fNIRS to study response inhibition tasks in children with ADHD aged 8 to 13 years and reported significantly lower activation in both the right and left DLPFC than in TD children. In contrast, Nakashima et al. used fNIRS in 14-year-old adolescents with ADHD during a multisource interference task and found that the left DLPFC showed hyperactivation relative to the control group.^61^ In addition, Dos Santos Afonso et al.^62^ observed that greater severity of ADHD symptoms was associated with increased activation in the left DLPFC among adults performing the Stroop task. These inconsistent findings suggest that lateralized differences in DLPFC activation may occur when individuals with ADHD engage in cognitive tasks such as the ST, highlighting a potential asymmetry in brain function related to ADHD. Generally, dysfunction in the left DLPFC is associated with deficits in working memory and cognitive flexibility, whereas dysfunction in the right DLPFC is related to inhibitory control.^63^^,^^64^ The results of the present study indicate that, regardless of ADHD severity, left DLPFC activation in the ADHD group may be flawed, suggesting that it could be a fundamental neurofunctional characteristic of ADHD. These findings underscore the need for further investigation of the neurofunctional characteristics of ADHD.

Correlation analyses between CAT performance and $\Delta {\mathrm{HbO}}_{2}$ during ST indicated that lower DLPFC activation during ST was associated with poorer CAT scores across groups. In the ADHD group, a significant negative correlation emerged only in the right DLPFC activation during ST, consistent with a lateralized contribution of the right DLPFC to attention and executive control. Notably, DLPFC activation during ST was related only to performance on complex attention tasks in the CAT; no significant associations were observed for simple attention tasks (e.g., selective auditory/visual attention). We suspect this pattern reflects our use of the Stroop paradigm to engage the cognitive control abilities and executive functions. These findings align with prior work showing greater DLPFC engagement during more demanding tasks and with models implicating the DLPFC in working memory, problem-solving, and cognitive regulation.^65^

Another important observation is the difference in attentional processing between children and adolescents. Chen et al.^66^ noted that although adolescents with ADHD often maintain good performance on simple attention tasks, they exhibit deficits in complex attention tasks, indicating that ADHD disproportionately affects tasks that require high-level executive function and complex cognitive processing. However, multiple studies have reported that children with ADHD struggle with both simple and complex attention tasks, whereas adolescents with ADHD primarily show deficits in complex attention tasks,^54^^,^^67^ which are related to brain development and maturation of executive functions. As the brain matures, children with ADHD who have difficulty paying attention develop into adolescents with ADHD, whose simple attention abilities have developed; however, difficulties with complex tasks that require advanced cognitive skills (e.g., working memory, planning, and inhibitory control) arise. Hong et al.^68^ found that deficits in divided attention and working memory persisted into adulthood in individuals with ADHD, highlighting the persistent nature of these challenges. Furthermore, Boen et al. demonstrated significant developmental progress in attentional processes from childhood to adolescence. Their findings suggest that executive control is fully developed by late adolescence, whereas directional awareness and vigilance continue to develop into early adulthood.^69^

Given the exploratory nature of our analyses, we do not claim a definitive developmental model. A speculative interpretation is that left–right DLPFC maturation could differ in adolescents with ADHD. The left DLPFC could reach a plateau earlier than the right, whereas the right might continue to mature into late adolescence. As a result, there may be age-related differences in lateralized brain activity and related cognitive functions between adolescents with ADHD and their TD peers. This account remains tentative and requires confirmation with longitudinal designs and larger samples. Considering the findings of this study, interventions for children with ADHD may benefit from addressing a broad range of attention processes, whereas interventions for adolescents with ADHD could focus on strategies for managing complex tasks. We caution against drawing clinical implications based solely on this research; it should be considered preliminary until further evidence accumulates.

Our study has several limitations. First, the findings were limited to an adolescent sample and may not apply to other age groups. Future research should secure larger samples through comparative studies involving individuals of all ages, from children to adolescents and adults, to examine the generalizability of these results. In addition, considering differences based on ADHD subtypes or sex is an important task for future studies.

Second, although the participants were verbally asked to stop their medication for the wash-out period, this was not verified by other means such as blood levels. There could be a chance that some participants may not adhered to the washout period as instructed.

Third, to gather ADHD participants without comorbidities, we checked for any condition other than ADHD in the past 12 months. Ideally, including only participants without any lifetime non-ADHD psychiatric disorders would have been best, but this was seen as unfeasible due to recall limitations. Therefore, participants who were diagnosed with a comorbid psychiatric illness but had reached remission for more than 12 months were included in the study.

Fourth, there were some methodological limitations related to our fNIRS data processing. Although the NIRSIT system incorporated 1.5-cm short-separation channels within the 204-channel dataset, the proprietary nature of the preprocessing pipeline precluded verification of whether proper short-channel regression methods, such as general linear model-based approaches, were implemented to remove systemic physiological interference from superficial tissue layers, including scalp blood flow, cardiac pulsation, and respiratory oscillations. Although band-pass filtering was applied, residual systemic artifacts cannot be completely ruled out without explicit short-separation regression. This uncertainty limits the interpretation of whether measured prefrontal activation reflects true cortical hemodynamic responses versus extracerebral contamination. Future validation using transparent analysis pipelines with documented short-channel regression procedures would strengthen the interpretation of prefrontal activation patterns in ADHD.

## 5 Conclusion

This study found that adolescents with ADHD exhibit distinct brain activation patterns in the DLPFC compared with the TD group during attention tasks. It demonstrates that fNIRS is an effective tool for measuring the differences in DLPFC activation between adolescents with and without ADHD. By focusing on adolescents rather than children, this study distinguishes itself from previous studies that predominantly explored ADHD in childhood, thus offering unique insights into the developmental trajectory of ADHD. In addition, this study provides empirical evidence using fNIRS, which supports existing findings that adolescent ADHD is associated with functional deficits in complex attention tasks. Because adolescence is a critical period of neurocognitive development, including the maturation of the prefrontal cortex, which continues into early adulthood,^18^ differences in DLPFC activation shed light on the neurobiological underpinnings of ADHD and offer valuable insights into its diagnosis and treatment. A thorough analysis of brain activation patterns using fNIRS can enhance our understanding of the neurofunctional characteristics of adolescents with ADHD and play a significant role in the development of effective therapeutic strategies. Moreover, the unclear activation patterns observed in the left DLPFC underscore the need for further research to better understand the cognitive difficulties experienced by adolescents with ADHD.

## Data Availability

The data that support the findings of this article are not publicly available due to privacy and ethical concerns. They are available from the corresponding author upon reasonable request.
